# Long COVID and Health Inequalities: What's Next for Research and Policy Advocacy?

**DOI:** 10.1111/hex.70047

**Published:** 2024-10-02

**Authors:** Sarah Akhtar Baz, Mirembe Woodrow, Donna Clutterbuck, Chao Fang, Jordan Mullard, Amitava Banerjee, Sarah Barley‐McMullen, Jd Carpentieri, Anne‐Laure Donskoy, Alice Faux‐Nightingale, Sasha Lewis‐Jackson, Margaret E. O'Hara, Tanvi Rai, Ondine Sherwood, Nina Smyth, Kirsty Stanley, Victoria Welsh, Ghazala Mir, Nisreen A. Alwan

**Affiliations:** ^1^ York Trial Unit, Health Sciences University of York York UK; ^2^ School of Primary Care, Population Sciences and Medical Education, Faculty of Medicine University of Southampton Southampton UK; ^3^ Sociology, Social Policy and Criminology University of Liverpool Liverpool UK; ^4^ Faculty of Medical Science Newcastle University Newcastle UK; ^5^ Institute of Health Informatics University College London London UK; ^6^ Long Covid SOS Charity Oxfordshire UK; ^7^ Institute of Education University College London London UK; ^8^ School of Sociology, Politics and International Studies University of Bristol Bristol UK; ^9^ Centre for Musculoskeletal Health Research, School of Medicine Keele University Keele UK; ^10^ Nuffield Department of Primary Care Health Science, Medical Sciences Division University of Oxford Oxford UK; ^11^ Long Covid Support Charity England and Wales UK; ^12^ Psychology, School of Social Sciences University of Westminster London UK; ^13^ Long Covid Kids Charity England, Wales and Scotland UK; ^14^ School of Medicine Keele University Keele UK; ^15^ School of Medicine, Faculty of Medicine and Health University of Leeds Leeds UK; ^16^ NIHR Southampton Biomedical Research Centre University of Southampton and University Hospital Southampton NHS Foundation Trust Southampton UK; ^17^ NIHR Applied Research Collaboration Wessex Southampton UK

**Keywords:** health inequalities, Long COVID, QLC network, qualitative

## Abstract

**Introduction:**

Organised by the ‘Qualitative Long Covid Network’, a workshop for qualitative Long COVID (LC) researchers, LC charity representatives and people with LC took place in June 2023, where research on the intersectional inequalities affecting LC prevalence, recognition and care was shared and discussed.

**Methods:**

Five key themes were drawn up from presentations, discussions and reflections during the workshop, which are presented in this study.

**Results:**

The following five themes are discussed: the unfairness of LC, difficulties in accessing care, mistrust of the healthcare system, a lack of understanding of LC and experiences of stigma and discrimination. Factors that widen or narrow inequalities related to LC were identified.

**Conclusion:**

A call to action is proposed to investigate and address inequalities through a robust LC research agenda that speaks with conviction to policy and decision‐makers. We argue that there needs to be a strong investment in research and evidence‐based policy and practice to mitigate the worst effects of the condition and address the inequalities in experience, treatment and support, which are experienced more often and more acutely by some of society's most vulnerable and disadvantaged individuals.

**Patient and Public (PPI) Contribution:**

Projects included in this article had PPI ongoing activity to inform their research. A member of the CONVALESCENCE PPI group presented at the QLC Network ‘Long Covid and Health Inequalities’ workshop, as did members of Long COVID Kids, Long COVID Support and Long COVID SOS charities. They were all invited to be co‐authors of this article.

## 1 Introduction

Long COVID (LC), also known as post‐COVID‐19 condition, is a multi‐system disease characterised by symptoms that continue or develop for weeks, months or years following SARS‐CoV‐2 infection. Symptoms include fatigue, breathlessness, chest pain, cognitive problems and cough among many others. According to the World Health Organisation's definition, these symptoms cannot be explained by an alternative diagnosis and have an impact on everyday functioning [1].

Research into intersectional inequalities affecting LC prevalence, recognition and care is in its early stages. An intersectional approach explores the multiple demographic, social, economic and health factors coming together to create disadvantages in health outcomes [2]. The need for a workshop to bring together key themes from various studies on the issue of LC and inequalities was conceptualised by G.M. and organised by the Qualitative Long COVID (QLC) Network in London in June 2023 to advance work in this area. Eighty‐six people registered at the hybrid workshop, which included talks from six qualitative studies (CONVALESCENCE [3], LC in Families [4, 5], HI‐COVE [6], LOCOMOTION [7], SPLaT‐19 [8] and STIMULATE‐ICP [9]), three LC charities (LC Kids, LC SOS and LC Support) and a patient and public involvement (PPI) representative from the CONVALESCENCE study. In the spirit of stimulating debate, research innovation and policy advocacy, we share key themes, reflections and recommendations arising from this workshop. Some studies' preliminary findings showcased in the workshop are presented here, with some already published and others awaiting publication.

## 2 Theme Development and Researcher Positionality

The joint first author (M.W.) took notes throughout the workshop to begin the process of developing key themes across the projects, referring to slide sets and a video recording for further detail and clarification where needed. Concepts that regularly occurred during presentations and discussions were noted and categorised by M.W. and then synthesised into five overarching themes. These initial themes were further developed by the workshop organising team (S.A.B., D.C., C.F., J.M., M.W., N.A.A. and G.M.) through feedback on written documents and discussion at regular online meetings, with all team members contributing to the drafting of the final manuscript.

Editing discussions were open, with all members encouraged to share ideas freely. Members were researchers working on LC projects who had an interest in health inequity and who were committed to co‐production with LC patients and the public. Senior members of the team were careful to ensure equitable relationships between authors, many of whom were early career researchers, supporting them to take lead roles in the workshop and manuscript development. Three members of the team had experienced LC themselves, which informed their knowledge of LC and analysis. All researchers were keen for their research to lead to more recognition of LC and to develop practical ways to address health inequity.

**Figure 1:**
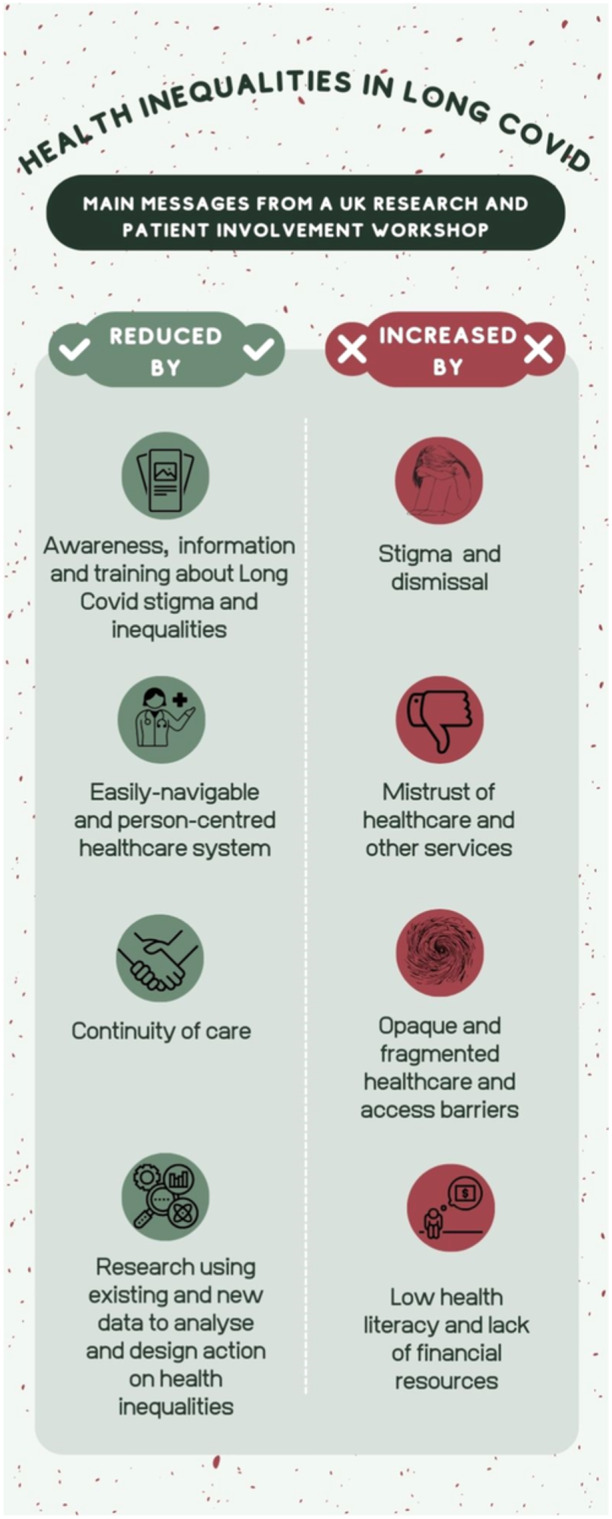
What potentially increases or reduces health inequalities in Long COVID.

Those who presented at the workshop, outside the organising team, are also co‐authors of this manuscript. They provided feedback and further edits on the draft manuscript. Final refinements were undertaken by the organising team, with N.A.A. developing Figure 1.

### 2.1 Theme 1: The Unfairness of LC

LC has multiple and diverse impacts on people's lives. Epidemiological studies show that the prevalence of LC differs across different population groups [10]. In the workshop, research teams reflected on the extent to which daily life is impacted by LC according to different intersectional characteristics. People from disadvantaged backgrounds often feel the greatest burden from LC, with limited access to information and resources increasing vulnerability (Theme 2).

In addition to physical effects, there are also emotional and psychological impacts. People's family lives and social roles can be severely impacted, including the education and prospects of young people with LC and the unexpected burden of new caring roles [11, 12]. There can be significant financial consequences, including loss of livelihood or careers, paying privately for healthcare (where affordable and where National Health Service [NHS] provision is unavailable or inadequate), early retirement, dietary changes and increased costs for childcare, transport use or home adaptations. These aspects of unfairness feature strongly in our multiple studies despite differing population samples and methods.

### 2.2 Theme 2: Difficulties and Barriers in Accessing Care

People living with LC report a range of difficulties in accessing primary and secondary healthcare. They report staff being uninformed about LC (Theme 4), not being believed or feeling neglected by their GPs or not being listened to by healthcare professionals [13]. People report inadequate advice and unaddressed healthcare concerns. Many LC support resources are only available online; this increases health inequalities potentially due to digital exclusion (access, literacy and confidence).

Intersectional inequalities in LC care and support relate to socioeconomic circumstances; for instance, accessing care may be more difficult for the self‐employed or those whose workplaces are inflexible [11]. Accessing care is also difficult for those with a disability, caring responsibilities and those less able to advocate for themselves. Navigating public transport to attend appointments can add further difficulty. Those with more privilege (e.g., experiences of navigating healthcare, from professional or White backgrounds) are better able to advocate for themselves.

Referral to secondary care or an LC clinic has been reported by patient groups as limited, and those from socioeconomically disadvantaged groups may be particularly under‐represented [14, 15]. Where referrals do take place, long waiting times and a high degree of persistence (a form of ‘emotional labour’) are needed for self‐advocacy [13, 16]. Consequently, many people develop their own treatment or self‐care programme. In some cases, medicines off‐prescription or healthcare services abroad without UK regulation may be sourced, often at own expense, putting patients at risk.

For people from disadvantaged groups, there are further difficulties in getting an LC diagnosis, and they may confront prejudice and discrimination (Themes 3 and 5) [16, 17]. There are differing opinions on the value of an LC diagnosis. Young people with LC, and their parents, found a diagnosis helpful for school, access to support, validation and recognition of people in similar situations. Those in employment also find a diagnosis helpful in negotiating a phased return to work. However, some felt that the LC label had become ‘a dustbin diagnosis’ [16]. Some healthcare professionals believed that diagnosis could even be a medical burden for patients. They avoided labelling, treating people according to their symptoms and using diagnosis strategically to facilitate treatments or services.

### 2.3 Theme 3: Mistrust of the Healthcare System

Against a backdrop of historical mistrust and fear of healthcare services among ethnic minorities [13, 16], there can also be intersectional experiences of mistrust in healthcare [13]. Some patients fear racial discrimination and being treated differently as an ethnic minority [6]. There are concerns reported about engaging with healthcare, including not seeing any benefit, and negative attitudes to help‐seeking. Racism within healthcare and employment creates further disadvantages.

People living with LC also express concerns about disclosing their symptoms, particularly psychological symptoms. Some participants in the STIMULATE‐ICP case‐finding study and LOCOMOTION were reluctant to engage with healthcare due to past experiences of physical symptoms being attributed to mental health conditions [17]. Some reported focusing on certain symptoms at the expense of others as the only way of sourcing the help needed. Those with underlying physical and mental health conditions or disabilities can struggle to be taken seriously.

### 2.4 Theme 4: Lack of Knowledge and Understanding of LC

Throughout the workshop, presenters and attendees reported a general lack of knowledge and understanding of LC variously among healthcare professionals, welfare system officials, the public and the media. Studies reported poor knowledge about the range of associated symptoms, the scale of the impact of the condition on individuals' daily lives and the treatment and support that is available. This may have significant negative implications for gaining a diagnosis, providing informed advice, making referrals for the right treatment and ultimately for patients' health.

Where people reported positive experiences of healthcare, professionals supporting them tended to have more understanding or were prepared to source further information. Better experiences—where people felt that their concerns were understood and validated and where tangible support was offered—were also often linked to an individual patient's greater health literacy and health system understanding.

Healthcare professionals' understanding of LC can impact access to wider healthcare, welfare services and further referrals; for example, welfare payments can be affected by deficient patient records.

### 2.5 Theme 5: Experiences of Stigma and Discrimination

Building on Theme 3, people living with LC feel that they may not be believed or that professionals do not believe that LC is ‘real’. Some people find difficulty sharing their experience of LC, with the role of family or community perceived as sometimes helpful and sometimes inhibiting. The ‘politicisation’ of COVID‐19 and LC induces scepticism, which creates an extra dimension of stigma unique to LC. In some cases, stigma prevented healthcare‐seeking behaviour or reduced social participation and increased reluctance to talk about LC [17].

Ethnic minorities with LC suspected racial discrimination played a part in the failure to believe their symptoms or referral to specialist support. Age was also a factor viewed as shaping experiences of discrimination; for instance, younger people feel dismissed, and older people may be told ‘it's your age’. Parents and children experience stigma [18] and inequity; for example, there is sometimes a perception that parents are ‘fabricating’ the illness, or young people worry that healthcare professionals mistrust test results [18]. Fluctuating symptoms and the extent to which symptoms were visible or diagnosed were further factors affecting patient credibility and the stigma they experienced.

There is evidence that positive healthcare experiences can occur for people with LC. Central to these experiences are continuity of care, validation and the feeling of being believed and listened to [19]. Although it is essential to address discrimination affecting all people with LC, findings on intersectional disadvantage highlight the need for additional initiatives to reduce age, disability, ethnicity and socioeconomic status.

## 3 Conclusion and Recommendations

This workshop drew together findings from several major studies exploring LC health inequalities in the UK context. Several factors were identified as capable of widening or narrowing inequalities in LC (Figure 1), and resultant recommendations have been drawn for research, policy and practice (Box 1 below).

Box 1 **Recommendations for research, policy and practice.**

Based on the workshop findings, the following are recommended for future research:

1. More collaboration between LC researchers, both in the United Kingdom and internationally
2. More emphasis on joined‐up research and early evidence synthesis to inform policymaking
3. Data sharing protocols that enable the removal of barriers to joining LC research (both qualitative and quantitative) and healthcare data
4. More emphasis on lived experiences and linking real people's stories with research findings
5. Research into the impact of LC on access to welfare payments and other governmental support
6. Studying how the inequalities experienced in LC compare to those of other LTCs

The following are recommended for policy and practice:

1. Greater recognition of LC and its wide spectrum of impact on individuals, so people are seen and heard, and stigma is reduced
2. Professional LC training across relevant services
3. Outreach and health promotion campaigns to increase understanding across wider society
4. Development of healthcare tools for LC that enable and empower patients in their healthcare journey and inform healthcare professionals
5. Routine intersectional analysis of healthcare data to identify equity actions for disadvantaged groups
6. Targeted initiatives that aim to reduce inequities in access to care and health outcomes
7. The use of multidisciplinary teams to treat LC
8. More consideration of LC within the context of care for other long‐term conditions; for example, considering the benefits and drawbacks of integrating LC specialist care and other long‐term condition care pathways
9. Better co‐development of peer support networks [20]
10. Continuation of funding for and development of specialised LC services that meet the needs of people with LC

*Note:* A ‘dustbin diagnosis’ describes where people received an LC diagnosis that wasn't welcomed because it was felt to be too broad and not necessarily tied to any specific treatment pathway that would meet their needs.

The similarities in findings across the studies represent a strength that provides weight to the themes presented in this study. Collectively, they highlight the range of experiences of LC and the importance of recognising the way multiple forms of disadvantage interact around the condition, generating very particular illness biographies with compounded disadvantage. Intersections of age, gender, ethnicity, socioeconomic status and disability all shape the experiences of the condition, and the healthcare and wider support sought and received. The findings are also relevant to other chronic conditions; further comparisons between LC and other LTCs will be useful to compare in terms of how inequalities are experienced.

Our combined inputs are timely given that SARS‐CoV‐2 remains a significant health risk. People with LC deserve recognition, and their perspectives are vital to shaping the response to this pervasive ongoing healthcare need. There is a real risk that LC is forgotten or shelved as a public health problem, particularly given the strong socio‐political drive to ‘move on’ from the pandemic. It is crucial that there is a strong investment in research and evidence‐based policy and practice that mitigates the worst effects of the condition and addresses health inequalities, which are often experienced by society's most vulnerable and disadvantaged groups. Despite our workshop taking place in June 2023, greater recognition of the condition—how it is experienced and how to address the health inequalities involved—is still needed in wider society, policy and practice 1 year later. Various research findings have been published since the workshop aim to contribute to achieving these outcomes [16, 17, 21, 22]. Further published findings from the studies discussed here will be key in advancing the evidence base in relation to reducing health inequalities in LC.

Throughout the workshop, participants reflected regularly on what needs to change in the policy and research sphere. We propose several recommendations and a ‘call to action’ as next steps for how concerns can be progressed through a robust LC research agenda that also speaks with conviction to policy and decision‐makers (Box 1).

## Author Contributions

**Sarah Akhtar Baz:** conceptualisation, writing–original draft, methodology, writing–review and editing, supervision. **Mirembe Woodrow:** conceptualisation, writing–original draft, methodology, writing–review and editing, data curation, formal analysis. **Donna Clutterbuck:** conceptualisation, writing–original draft, writing–review and editing, methodology. **Chao Fang:** conceptualisation, writing–original draft, writing–review and editing, methodology. **Jordan Mullard:** conceptualisation, writing–original draft, writing–review and editing, methodology. **Amitava Banerjee:** writing–review and editing. **Sarah Barley‐McMullen:** writing–review and editing. **Jd Carpentieri:** writing–review and editing. **Anne‐Laure Donskoy:** writing–review and editing. **Alice Faux‐Nightingale:** writing–review and editing. **Sasha Lewis‐Jackson:** writing–review and editing. **Margaret E. O'Hara:** writing–review and editing. **Tanvi Rai:** writing–review and editing. **Ondine Sherwood:** writing–review and editing. **Nina Smyth:** writing–review and editing. **Kirsty Stanley:** writing–review and editing. **Victoria Welsh:** writing–review and editing. **Ghazala Mir:** conceptualisation, writing–original draft, methodology, supervision, writing–review and editing. **Nisreen A. Alwan:** supervision, writing–review and editing, writing–original draft, conceptualisation, methodology, visualisation.

## Ethics Statement

No additional specific ethical approval was required for this piece of writing, as it involved no additional data collection and is a write‐up of presentations and discussions from a workshop organised by the Qualitative Long Covid Network. Each respective project involved has obtained ethical approval; details can be found in the relevant publications listed here.

## Conflicts of Interest

The authors declare no conflicts of interest.

## Data Availability

Data sharing is not applicable to this article as no new data were created or analysed in this study.
